# EFFECTIVENESS OF MULTICOMPONENT EXERCISE INTERVENTIONS IN DEMENTIA PATIENTS: A SYSTEMATIC REVIEW

**DOI:** 10.1093/geroni/igz038.3317

**Published:** 2019-11-08

**Authors:** Oscar Ribeiro, Flavia Borges-Machado, Nádia Lima, Paulo Farinatti, Joana Carvalho

**Affiliations:** 1 University of Aveiro - Department of Education and Psychology | Center for Health Technology and Services Research (CINTESIS.UA), Aveiro, Portugal; 2 Research Centre in Physical Activity, Health and Leisure, Faculty of Sport Science, University of Porto, Porto, Portugal; 3 Laboratory of Physical Activity and Health Promotion, University of Rio de Janeiro, Rio de Janeiro, Brazil

## Abstract

Multicomponent training (MT) combines aerobic, strength, postural, and balance exercises, and has been presented as a promising intervention strategy for dementia due to its potential influence in treating symptoms and/or delaying the disease progression in addition to its intrinsic health benefits. This study aims to systematize evidence on how effective MT is in what concerns physical fitness, cognition, and functionality in dementia patients. Four databases (PubMed, WoS, Scopus & SportDiscus) were systematically searched to locate potential trials through March 2019. A total of 2,312 records were identified and a final set of 17 manuscripts reviewed; of these, only 6 satisfied all inclusion/exclusion criteria: peer-reviewed studies performed with humans aged 60+ years; interventions exclusively MT conducted with clinically diagnosed dementia patients; controlled trials (randomized or not). Cochrane Collaboration’s tool was used to estimate risk of bias. Samples sizes ranged from 27 to 170 participants; MT programs lasted between 4 weeks up to 12 months, took place from a daily basis to twice a week, and sessions ranged from 30 to 60min. Routine medical care was the most frequent intervention offered to control groups. In overall, studies revealed that MT benefits agility/balance, gait speed and strength. Evidence remains unclear regarding MT effectiveness on increasing cognitive function and ADL performance, although maintenance and more pronounced decline on control groups were reported. MT may be an important non-pharmacological strategy to enhance physical conditioning on dementia patients, but further evidence is needed for acknowledging its benefits in specific cognitive abilities and ADL performance.

